# Women’s body image after mastectomy: a photovoice study

**DOI:** 10.1007/s00520-025-09541-3

**Published:** 2025-05-27

**Authors:** Yasemin Erden, Hatice Cecen Celik, Nurgül Karakurt

**Affiliations:** 1 Nursing Department, Faculty of Health Sciences, Erzurum Technical University, Erzurum, Turkey; 2 Department of Emergency Aid and Disaster Management, Faculty of Health Sciences, Erzurum Technical University, Erzurum, Turkey

**Keywords:** Body image, Photovoice, Cancer, Mastectomy

## Abstract

**Purpose:**

Breast cancer is defined as a severe chronic disease commonly seen in women. In addition to the anxiety and fear of cancer, breast cancer can lead to the loss of an essential symbolic image for women. This study aims to reveal the emotional experiences of women who have undergone mastectomy surgery during and after the disease process and to evaluate the impact of mastectomy on body image using the photovoice method.

**Methods:**

The research group consists of fifteen women patients who underwent mastectomy and received treatment for breast cancer in the chemotherapy unit of a hospital in eastern Turkey between 2022 and 2023. Data were collected through photographs and semi-structured interviews.

**Results:**

The psychological, emotional, physical, and social problems of individuals who have undergone mastectomy were analyzed along with their photographs and explanations. Fifteen women aged between 45 and 65 who had undergone mastectomy surgery participated in this study, resulting in 25 photographs depicting their experiences. Of these, 12 photographs were analyzed in stages, considering comment richness and maximum diversity. Thematic analysis was used to analyze the data. As a result of the analyses, three main themes are isolation and feelings of inability to cope, the importance of psychosocial support and religious beliefs, and perceptions regarding external appearance: loss and grief, and eight sub-themes under these main themes.

**Conclusion:**

The analyses conducted have shown that the loss of an important organ representing femininity due to mastectomy surgery has a significant impact on body image and consequently brings about many psychosocial problems.

## Introduction

Breast cancer emerges as a significant global public health concern affecting women, ranking as the most prevalent form of cancer, particularly surpassing lung cancer. Annually, over 2.1 million women receive a diagnosis of breast cancer worldwide [1]. In Turkey, the incidence stands at 40.7 per 100,000 women, with approximately 15,000 new cases reported each year [2]. Technological advancements and improved early detection mechanisms have notably contributed to enhanced survival rates among breast cancer patients, leading to the formation of a substantial cohort of breast cancer survivors [3]. Various treatment modalities are employed for breast cancer based on disease staging, with surgical intervention being a prominent approach. Surgical procedures, predominantly mastectomy or breast conservation, are undertaken to either control cancer cell proliferation or mitigate cancer risk. Mastectomy, encompassing the removal of breast tissue, is performed in diverse forms depending on individual patient circumstances.

Nevertheless, it signifies the relinquishment of a symbolically and identity-integrated organ in the context of breast cancer. Despite its efficacy in health management, mastectomy can instigate significant alterations in body image, thereby causing women to grapple with the loss of an organ emblematic of femininity and sexuality [4]. Such ramifications extend beyond the physical realm, permeating into emotional spheres, thereby underscoring the multifaceted impact experienced by women undergoing mastectomy. The repercussions of breast surgery on women can be as profoundly distressing as the experience of cancer itself, particularly when viewed through the lenses of body image and self-esteem. This underscores not only the physical but also the psychological dimensions of the trauma endured by women [5].

Body image constitutes a psychological construct entailing individuals’ perceptions, sentiments, and attitudes toward their bodies. Tangible shifts in women’s body image perceptions have been associated with adverse psychological consequences [6, 7]. Bagheri and Mazaheri [6] noted a marked discrepancy in body image and quality of life between female breast cancer patients and their healthy counterparts. Furthermore, an altered body image has been identified as a factor contributing to depressive symptoms within the initial 5 years following a cancer diagnosis (Aguado [8]). Similarly, Luutonen et al. [9] explored the psychological implications of breast cancer, revealing that 32% of participants exhibited depressive symptoms, while 28% experienced emotional distress. The loss of breasts for women carries connotations such as the loss of femininity, the cessation of sexuality, and the sense of incomplete motherhood. Following the initial shock and denial, these concerns become more pronounced, complicating the adaptation to the disease and its treatment [10]. Research indicates that negative alterations in body perception post-mastectomy disrupt sexual satisfaction and marital harmony [11, 12]. Wang et al. [13] categorized seven primary themes associated with sexual well-being among 20 Chinese women post-breast cancer treatment, encompassing a decline in sexual frequency, diminished sexual interest, menopausal symptoms, changes in body image, impacts on marital relationships, misconceptions about sex, and the necessity for professional consultation. In light of this collective body of evidence, this study aims to evaluate the perspectives and perceptions of women who have undergone mastectomy concerning body image, utilizing the photovoice methodology.

## Photovoice

In the past two decades, health researchers have employed various visual research techniques [14]. These include methods such as photo elicitation [15], participant photography [16], and photovoice [17]. These approaches rely on photography as a central tool for collecting data on health and illness. They enable engaging conversations with participants during interviews and sharing data with broader audiences, such as through community exhibitions. When integrated with narrative methodologies, photography has demonstrated effectiveness in elucidating the first-hand experiences of individuals regarding health and illness. Researchers have applied visual techniques to explore embodied encounters with conditions such as cancer [18] and dementia [19].

Photovoice is one of the most commonly used and extensively replicated visual methodologies in health-related research [20]. Its creators describe it as a powerful social research tool that empowers marginalized groups by enabling them to document their experiences and become potential agents of change within their communities [17], p. 369. While photovoice has found application in diverse settings [21], it has been comparatively less utilized in the realm of women’s health [22]. This research was conducted using the photovoice method to gain a more comprehensive understanding of the impact of mastectomy on women’s body image.

## Methods

### Research design

Photovoice is a widely used visual method in health research that empowers marginalized groups to document their experiences and advocate for change. Although applied in various contexts, it remains underutilized in women’s health. This study adopts photovoice for its strength in fostering participant collaboration and capturing emotional depth, aiming to generate richer visual and narrative insights.

### Data collection

This study was conducted qualitatively using the photovoice method to explore the body image perceptions of individuals who had undergone mastectomy. Participants were asked to take photographs that they believed represented their body image. In this context, individuals were encouraged to capture visual representations based on their personal life stories and experiences using their own mobile phones. The visual data produced by the participants were digitally submitted to the research team via email. This process was designed with careful attention to participant confidentiality and adhered to the principle of voluntariness. In doing so, a participant-centered data collection approach was adopted, allowing participants to express their personal experiences through visual narratives.

Study involved the participation of fifteen women aged 46–65 who had undergone mastectomy and were still having chemotherapy. During recruitment, women were briefed about the study, and written consent was obtained. Subsequently, participants engaged in one-on-one sessions with the researcher to discuss their photographs. This setting allowed the researcher to ask individual participants questions about their initial data analysis. For instance, it served as a platform for discussing sensitive topics related to the phenomenon of interest. The following questions were posed to understand better how social, cultural, or contextual factors intersect with the phenomenon of interest. What do you see in this photo? What do you want to tell us in the picture here? How does this photo relate to your current body image? Would you like to share your feelings and thoughts about your body after mastectomy surgery? Are you considering having a breast prosthesis in the future? What advice would you give to a woman who will have a mastectomy?

The interview schedule was applied with considerable flexibility, allowing for the expansion of discussions to cover new topics or the avoidance of subjects that the patient found particularly challenging (with the assessment and sensitivity of the interviewer guiding this decision). Throughout the interview, researchers adjusted to the woman’s readiness to continue or pause the discussion, to respond or withhold. Striving for empathy and authenticity in the approach, researchers adjusted based on the women’s readiness or hesitancy to share personal information during emotional turmoil. This sample size aligns with the quality standard of saturation, ensuring a sufficient level of depth and breadth in addressing the topic [23]. Each interview ranged from 30 to 60 min, and thorough verbatim recordings were made and transcribed. Despite the challenging circumstances the women were undergoing during the interviews, they all conveyed appreciation for the opportunity to discuss their feelings and share their experiences openly.

### Data analysis

This study utilized data from both participant-taken photographs and related interviews. The thematic analysis adhered to the approach proposed by Braun and Clarke [24], employing an inductive method where themes emerged organically from the data rather than fitting within a predefined framework. The process followed the five phases outlined by Braun and Clarke [24]: (i) becoming familiar with the data, (ii) generating initial codes, (iii) searching for themes, (iv) reviewing themes, and (v) defining and naming themes. The authors transcribed the audio recordings and reviewed the manuscript to discern patterns. Marginal notes were incorporated into the document to create initial codes, summarizing the fundamental meaning of the data. A thematic map was then constructed to organize and explore the relationships between codes, identifying main themes and sub-themes. Finally, the themes were refined, and names capturing the essence of each theme were assigned. The data analysis was conducted manually, by Braun and Clarke’s recommendation, and no software package was employed for this purpose.

Various strategies were adopted to ensure the methodological reliability of the data analysis. A single researcher did not carry out the analysis process; instead, a team-based approach was chosen to reduce potential subjectivity and enhance analytical depth. Two independent researchers coded the data separately and then compared their coding processes to assess inter-rater consistency. Any discrepancies in coding were resolved through mutual discussion, thereby strengthening the reliability of the findings. In addition, an audit trail documenting all analytical decisions was maintained to ensure the transparency and traceability of the research process. All these practices significantly contributed to the reliability and methodological validity of the analysis phase of the study.

### Ethics

The study obtained ethical approval from the University Hospital Ethics Board, and all participants in the research were obligated to furnish written informed consent after the researcher provided detailed information about the study’s purpose and design. The study adhered to the Declaration of Helsinki and it was structured to ensure that participants would not undergo any harm.

## Findings

Fifteen women aged 45 to 65 who had undergone mastectomy surgery participated in this study. The number of participants was determined as 15 upon reaching data saturation. Patients described their body images after mastectomy through photovoice, addressing three main themes: isolation and feelings of inability to cope, the importance of psychosocial support and religious beliefs, and perceptions regarding external appearance including loss and grief. In total, eight sub-themes were identified under these overarching categories.

### Theme-1: Isolation and feelings of inability to cope

Within this framework, patients’ perceptions regarding body image following mastectomy will be elucidated, outlining four distinct subcategories: withdrawal from crowded settings, inclination towards solitude, challenges in maintaining productivity, and a conspicuous deficiency in vitality and motivation concerning fulfilling responsibilities. Furthermore, patients have underscored the notable prevalence of concepts such as emotional distress, diminished hope, fatigue, inclination to avoid external stimuli, and inward focus.

**Fig. 1:**
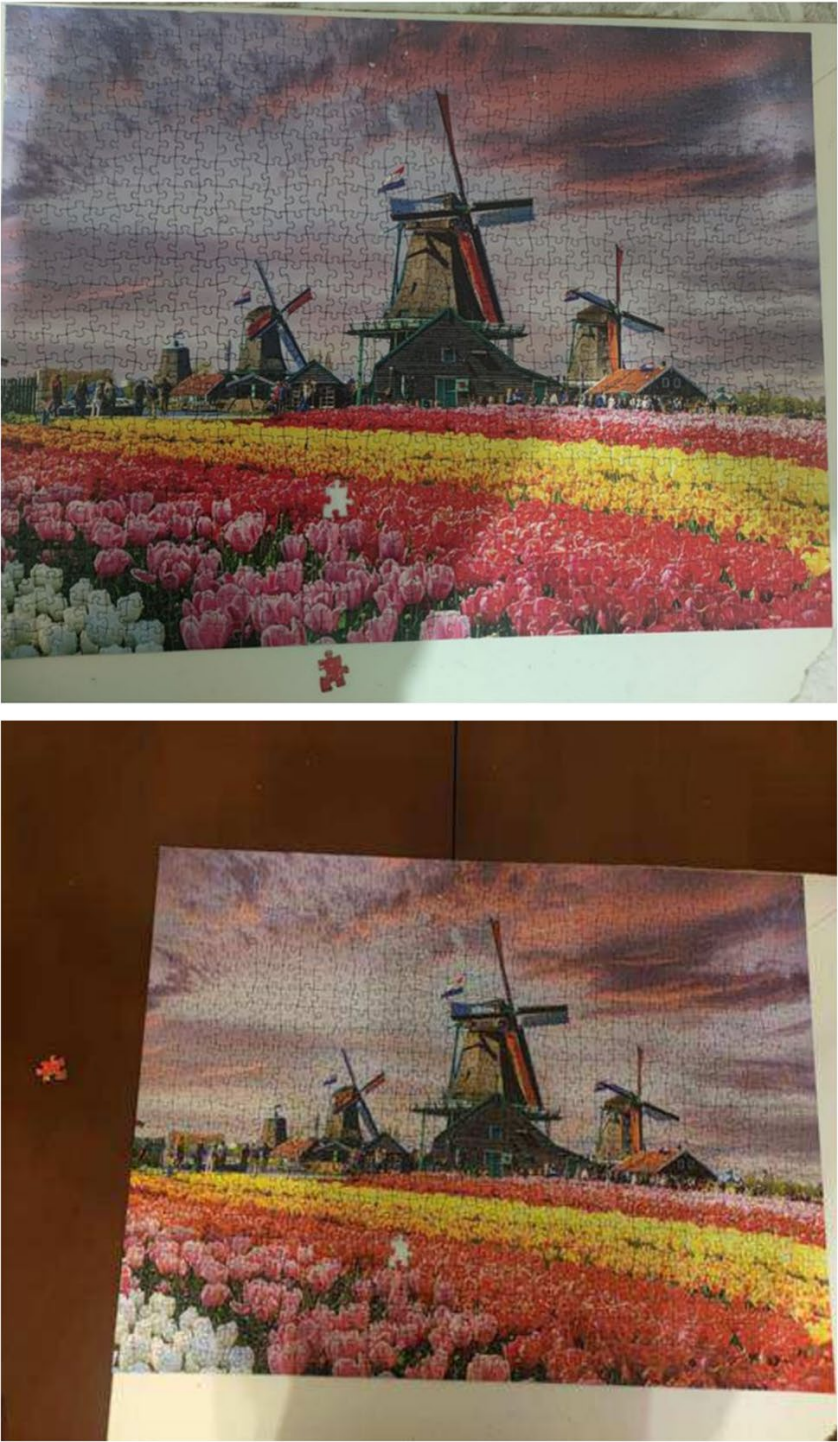
Being isolated

Figure 1 illustrates a puzzle, with a notable absence of one puzzle piece. Within this imagery, the patient depicted a sensation of being detached amidst a crowd. They conveyed a perception that those surrounding them could not fully grasp their lived experience, underscoring the uniqueness of their health challenges and the ongoing therapeutic regimen. The patient articulated that genuine understanding could only come from individuals who have shared a similar journey, thus accentuating feelings of isolation and the inherent challenge of communicating their circumstances to those lacking comparable experiences.

Patient 1: There are so many people around me, just like the pieces of this puzzle, but I feel utterly alone….. Yes, plenty of people are around me, but none truly understand me. They don’t know the fires burning inside me, the experiences I’m going through. Everyone around me says they understand, but someone can’t grasp this process unless they’ve been through it themselves. They try to cook for me and clean the house, in reality, they know I no longer have my former strength. But I don’t want to do cleaning or cooking; I want to be listened to and understood without them saying, “I understand you.”

Patient 5: After this illness, my family and relatives, bless their hearts, did not leave me alone. However, it saddens me greatly when they fail to understand every time I am upset or crying, saying things like, “What more do you want? Look, one breast is gone, but thank God you are healthy; you will get better.” It hurts me deeply that they do not understand. It’s not just my breast that’s gone; I have no hair, no eyebrows, not even my old strength. If I try to do any work, my arm swells up instantly. Yet, they make me cry by saying, “You are fine.” They say they understand but cannot truly grasp it unless they have experienced it. Sometimes, I think, “I wish I had no one; I am alone in a crowd.”

**Fig. 2:**
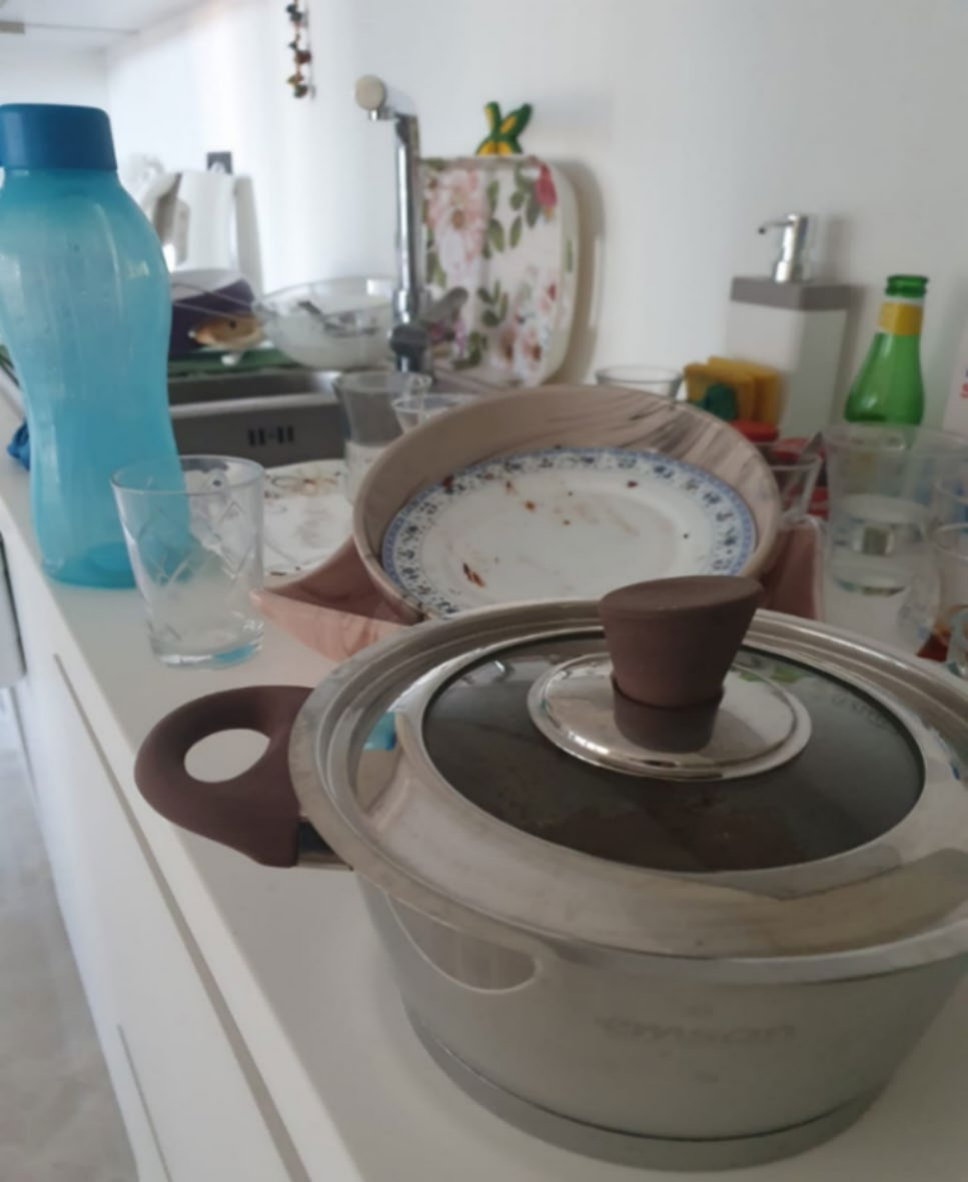
Incapability

In Fig. 2, a depiction of a cluttered household is presented. The patient utilized this imagery to illustrate the diminishment of their physical vitality and vigor, emphasizing that post-surgery, particularly since initiating chemotherapy, their bodily capabilities have undergone considerable alteration. The patient articulated experiencing profound weakness throughout the treatment process, highlighting a marked decline in their physical resilience through this visual representation. When attempting household chores, the patient delineated the challenges faced, particularly referencing the discomfort and swelling in the arm corresponding to the side of the breast removal surgery. The escalating disorder within their environment, coupled with the inability to accomplish tasks, prompted the patient to underscore feelings of ineffectiveness.

Patient 3: There are many tasks at home, but I cannot do them. I get tired quickly, and I become weak immediately. Besides, I cannot go to work anymore. I had to leave my job. I used to work as a cleaning staff, but unfortunately, I cannot do my job anymore. I cannot keep up with the housework; I have no strength to do any task. There is much clutter around me, but I cannot handle any of it. I have two children; one is away in another city, and the other is in the military. I do not know how things will be when he returns. We have not told him anything; I cannot bear to see him upset. I want to get back on track and do my job as I used to before he came, but I do not know how it will be possible.

Patient 6: After my breast was removed, all the responsibilities around me fell apart. I cannot keep up with anything; I used to care for both work and my children easily. However, now, everything is scattered, and when I try to tidy up, my arm swells up, and there is an unbearable pain under my armpit. My body no longer feels like mine; it is fragile and powerless. After the treatment, especially following chemotherapy, I become even weaker. I do not know if I can return to my old self after the treatments are over.

**Fig. 3:**
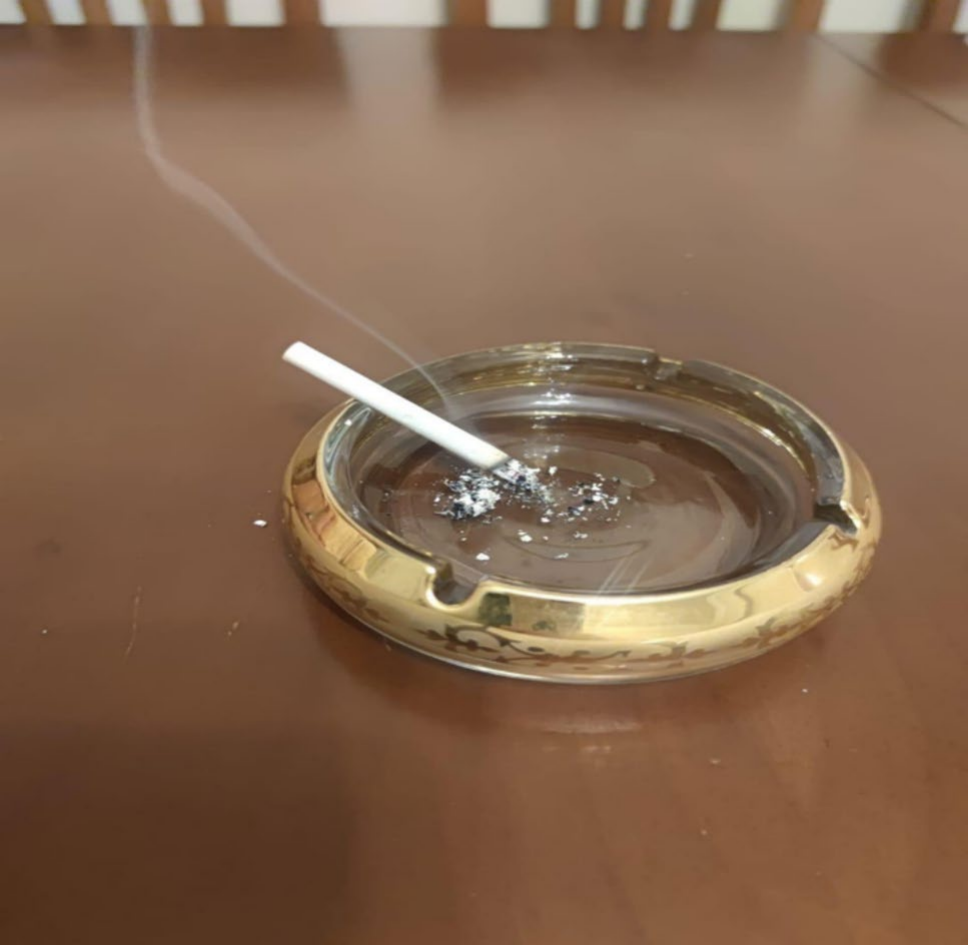
From non-existence to existence

In Fig. 3, the patient elucidated a coping mechanism involving the evasion of their present circumstances and the alterations in their physical appearance (such as hair and eyebrow loss and breast removal) by resorting to smoking. Despite receiving counsel from those in their surroundings to prioritize self-care, attend to their health needs, and exhibit mindfulness in their dietary and drinking habits, the patient disclosed a heightened inclination towards smoking, intensifying their smoking habits. They underscored experiencing a sense of amelioration while engaging in smoking, thus accentuating their proclivity to withdraw from social interactions and find solace in smoking**.**

Patient 4: There are so many people around me, all saying something, advising me not to do this or that, but I do not listen to anyone. I want everyone to leave me alone so I can quickly light up a cigarette. No one knows that I smoked during the treatment; I did not smoke then, but I lost my closest friend at that time, as if I lost a part of myself. Then I started immediately. I only feel good when I am smoking. My hair is falling out, my eyebrows are gone, spots appear on my face, my arm swells, and sometimes the pain becomes unbearable. Of course, my breast is gone; I have transformed into something other than myself. I do not recognize the woman in the mirror, but when I’m smoking, I confide in my cigarette. It has become my close friend.

### Theme-2: The importance of psychosocial support and religious beliefs

In this theme, patients’ post-mastectomy body perceptions are explained by three subsidiary themes. Patients underscored the paramountcy of social support, the relevance of intra-familial communication, and the significance of prayers and religious observances. Conspicuously, prevailing sentiments among the patients encompassed hope, endeavors toward recovery, and an overarching sense of trust and acceptance.

**Fig. 4:**
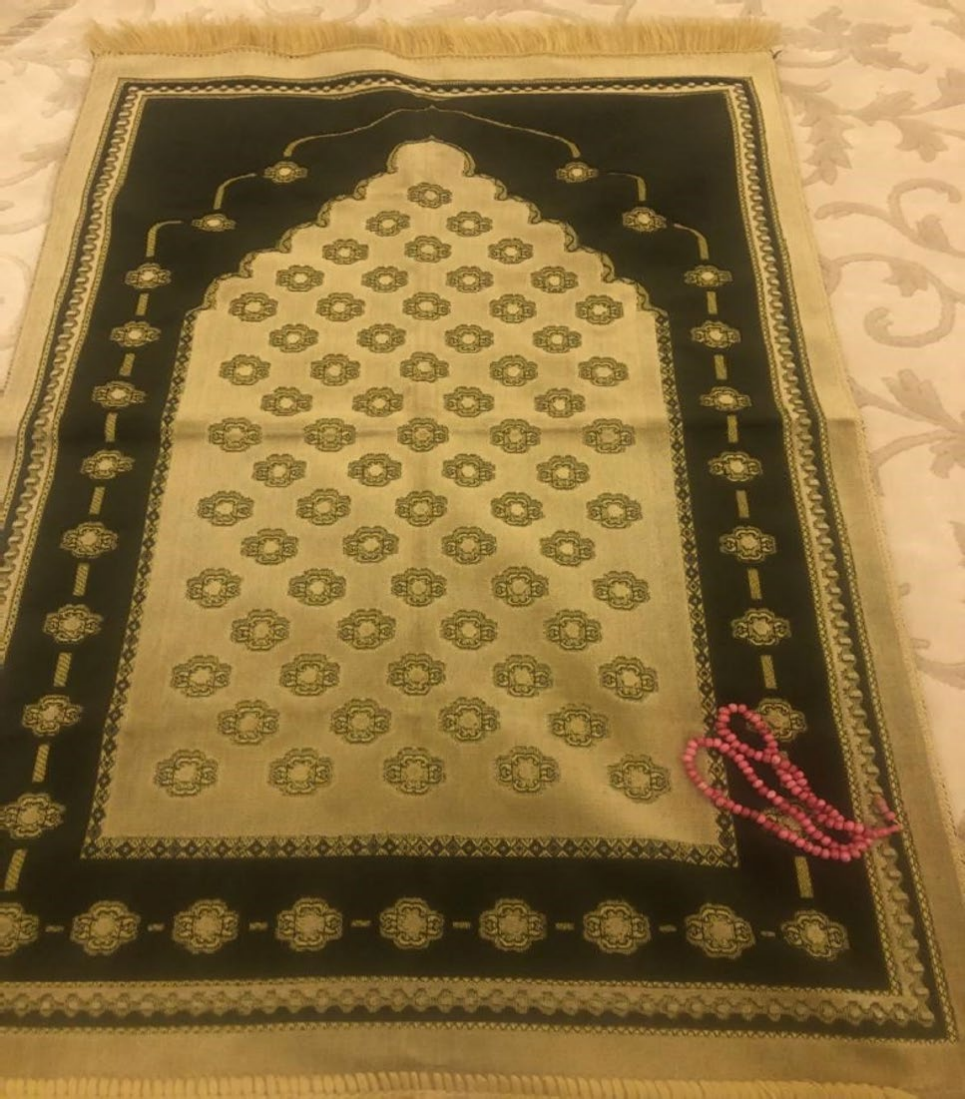
Spiritual strength

In Fig. 4, the patient deliberated on the importance of prayer and religious devotion in navigating illness and shaping body perception. They articulated that praying and extending their hands in supplication offered solace and comfort in moments of vulnerability throughout the disease journey. The patient underscored that worship and prayer fortified their resilience and upheld their resolve during adversity.

Patient 2: After my breast was removed, I turned only to Allah; I sought help only from Him because I knew that only He could help me. I feel very relieved when I pray.

Patient 7: The only thing that comforts my soul is my prayers. Sometimes I think, what if I did not have faith? Who would I ask for help then? Praying brings me much relief. I believe that everything will be fine with God’s will. Everything comes from Allah. I do not know what is good and what is bad. Surely, there is goodness in what I am going through. I will live, be patient, pray, and hopefully see it, God willing.

**Fig. 5:**
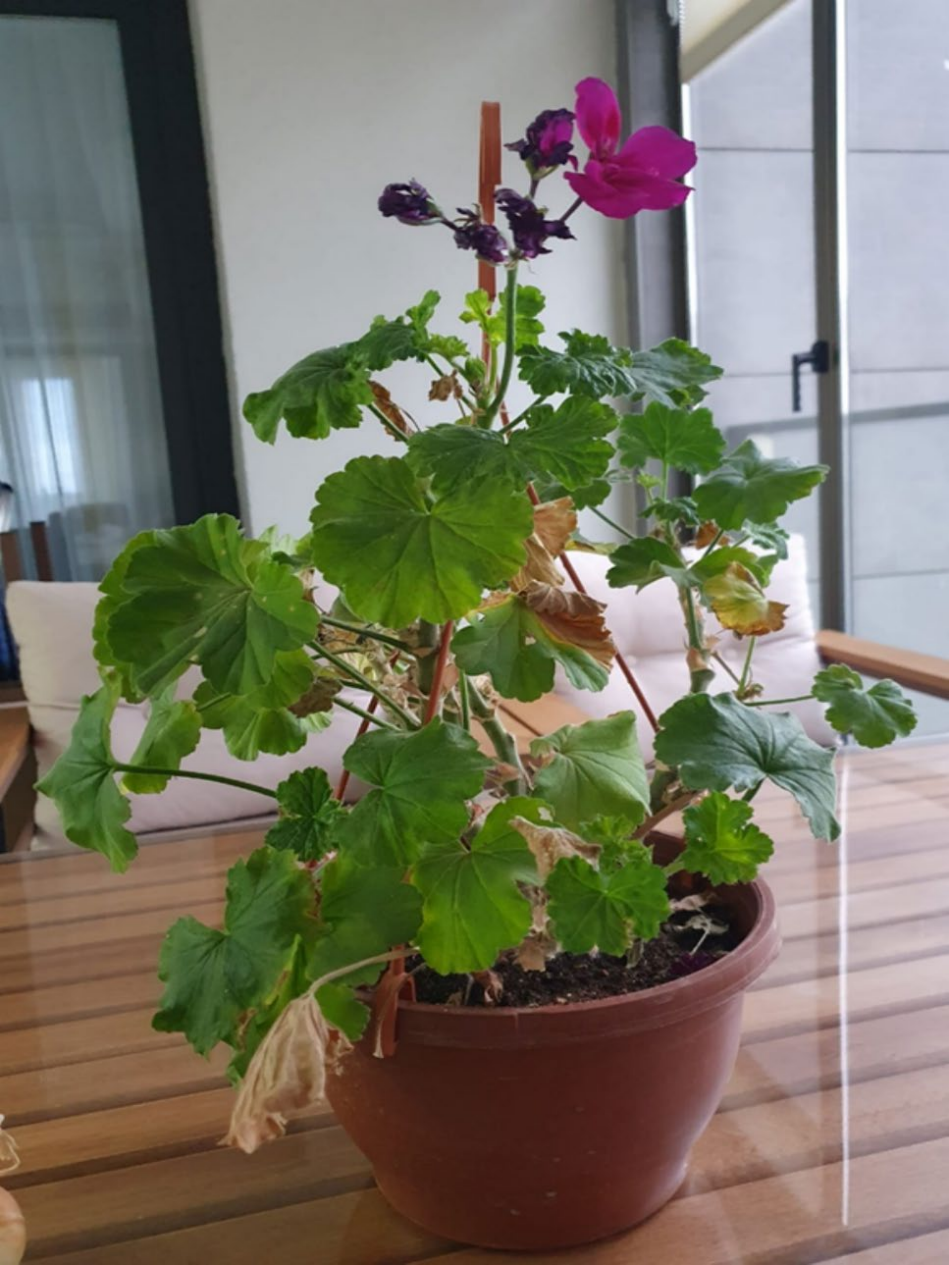
Rebirth

In Fig. 5, the patient employed a floral representation to symbolize their body image. They sought to convey that with the bolstering support of their familial network, particularly their spouse and children, they experienced heightened contentment and inner peace. The patient disclosed that they traversed the journey with greater ease due to the unwavering backing of their family, utilizing the flower metaphor to illustrate both the progression and the scars on their body. They underscored that the joy and serenity within their family, particularly witnessing their spouse’s and children’s happiness, surpassed the significance of their adversities. The patient articulated aspirations of resurgence akin to the depicted flower blossoming anew, underscoring the profound influence of witnessing familial happiness on their rejuvenation.

Patient 8: As long as my children and my spouse are with me and we are peaceful and happy, the removal of my breast is not important. The removal of my breast and the loss of my hair initially affected me a bit, but it affected me even more when my children heard about it and felt sad. Now they have gotten used to it, and when they are fine, I am fine too. Now, I am continuing my life in the same way. My children have adapted, and when they are happy, I am even happier.

Patient 10: When I first heard about this illness, my immediate concern was my children. I wondered how to tell them what they would say and if it would make them sad. When I got home, I told my spouse, “Let us go out to eat together; let’s go to the kids’ favorite place, and we can tell them there.” They cried a lot when they heard about it, and my heart was broken. But then, they got used to it and were always by my side. Yes, my breast was removed, a part of my body was gone, and my hair and eyebrows fell out, but I never felt sad so that my children would not be sad. After my breast was removed, I sat down and comforted my children. My body is for my children; if they are happy, what more could I want.

**Fig. 6:**
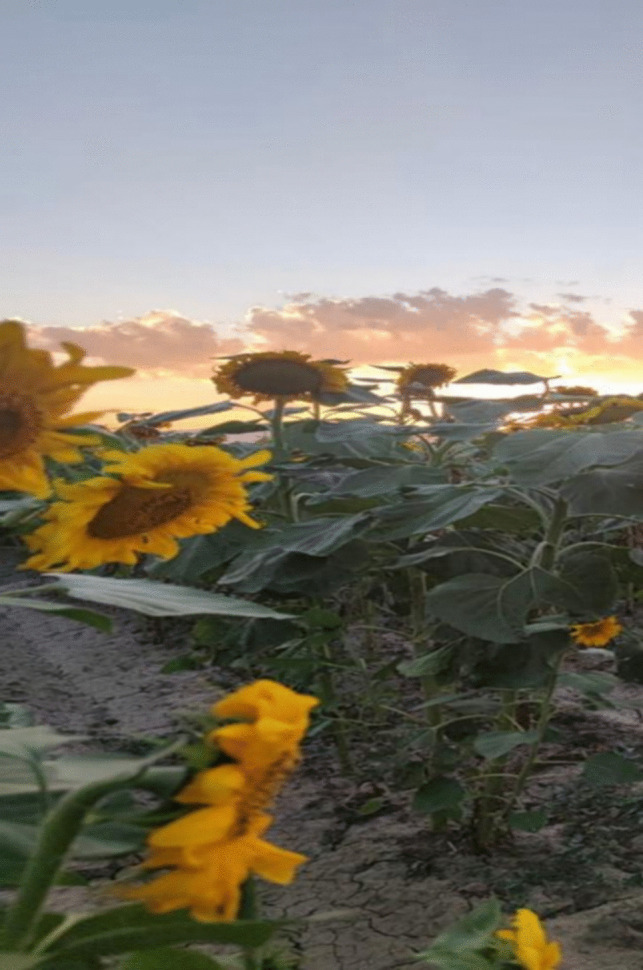
Not giving up

In Fig. 6, the patient portrayed their body image as shrouded in dark clouds, juxtaposed with a depiction of rejuvenation represented by sunflowers, emblematic of the rebirth of sunlight. Despite encountering adverse alterations in their physique following mastectomy, hair loss, and eyebrow loss due to illness, the patient elucidated their process of renewal and resurgence, buoyed by the unwavering support of their family, spouse, and children. The imagery encapsulated the patient’s narrative of resilience and revitalization facilitated by the steadfast presence of their loved ones.

Patient 11: When I first heard about my illness, I did not believe it. We went to many doctors, and I thought it could not be true. It felt like dark clouds descended upon me. Then, when I found out it was true, they immediately said my breast needed to be removed. Since that day, my spouse, children, and family have not left me alone. They cared for me like a child. They did everything to make me happy. My children do not know everything they do to keep from upsetting me. Yes, my breast was removed, and I was very sad. I lost my hair and eyebrows, and I have much pain. But, thanks to my children, spouse, and family, I stood up again. If it were not for them, what would I have done? That is when I would have fallen apart. That is when I would not have been able to escape from this illness. Their efforts kept me standing. They once again showed me how important family is. Thanks to them, I am here.

### Theme-3: Perceptions regarding external appearance: loss and grief

Patients’ post-mastectomy body perceptions are explained in this theme, elucidating five subsidiary themes under the overarching category of perceptions related to external appearance: loss and grief. Within this thematic framework, they underscored concepts such as loss, grief, identity crisis, feelings of incompleteness, and diminished self-worth. The alterations in their external appearance, notably attributed to breast removal, precipitated a profound shift in their sense of identity, rendering patients unable to reconcile their self-perception as women. They highlighted a perception of resembling masculine traits in their outward appearance. The surgical removal of the breast, coupled with the loss of hair and eyebrows during treatment, evoked a deep-seated sense of mourning and sorrow. Moreover, the physical exhaustion experienced by patients precipitated a significant emotional downturn, culminating in notable psychological distress.

**Fig. 7:**
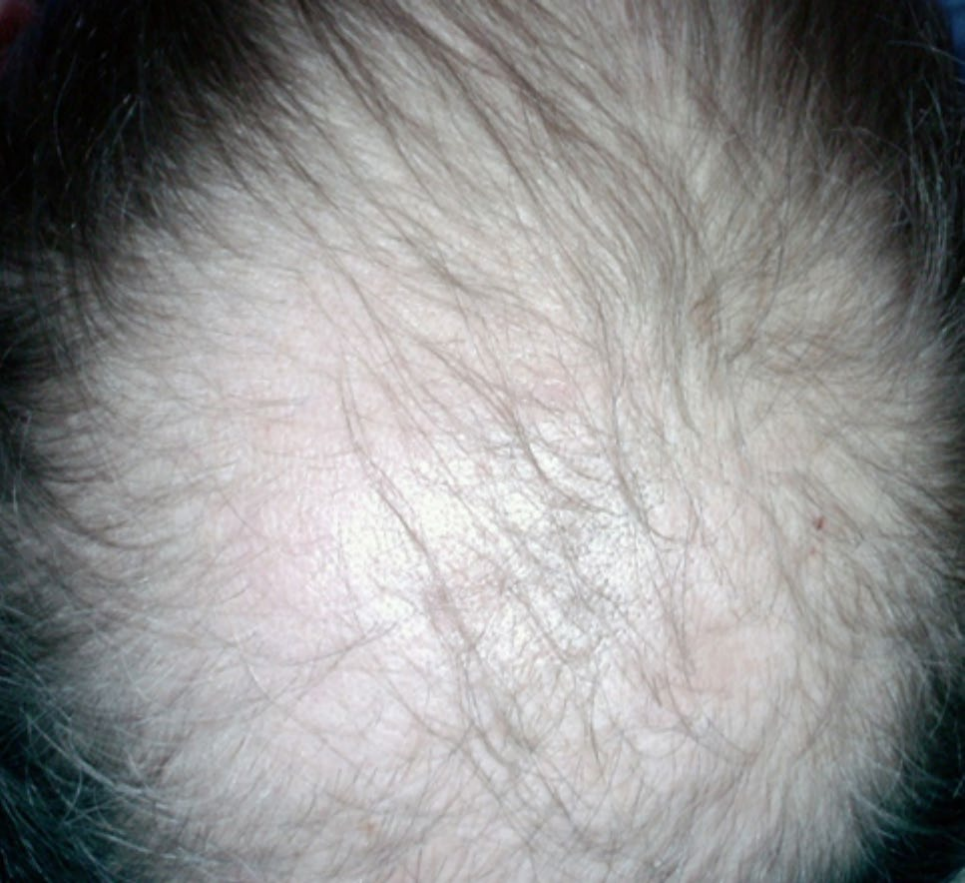
Making peace with change

In Fig. 7, the patient symbolically represented their body image through an illustration depicting a person experiencing hair loss. In addressing the topic of body image post-mastectomy, the patient underscored the paramount significance of their hair loss, particularly emphasizing this aspect through the chosen imagery. The patient conveyed their perception that hair loss following mastectomy holds greater weight than the surgical removal of the breast. They articulated that the visible shedding of hair has a more pronounced impact on their emotional state then the absence of the breast. While acknowledging the initial shock associated with mastectomy, the patient remarked that concealing the breast with clothing is relatively manageable. However, they emphasized that the conspicuous loss of hair and the absence of eyebrows, evident each time they confront their reflection, exert a more substantial and deleterious influence on their mood.

Patient 5: After the surgery, my breast was removed, and later, when the treatment started, my hair and eyebrows fell out. Even my skin color changed, and I got spots on my face and body. I do not feel like a woman anymore; I feel like someone else, like a man when I look in the mirror. It is as if I am looking at another person in the mirror, and I do not want to look in the mirror.

Patient 9: After my breast was removed, having a space there was tough, but I covered it with clothes. Sometimes, I put cotton in my bra so that it is not noticeable. When my hair and eyebrows fell out, it made me forget about my breasts. Everyone advised me to cut my hair before starting treatment, but I did not listen to anyone and did not cut it. I wish I had cut it; my hair fell out one by one into my hands. When I touched any strand of hair, it came out in handfuls. Then, my eyebrows disappeared, and the color of my skin changed. There is no trace of my old self after the treatment; returning to my old self is not possible.

**Fig. 8:**
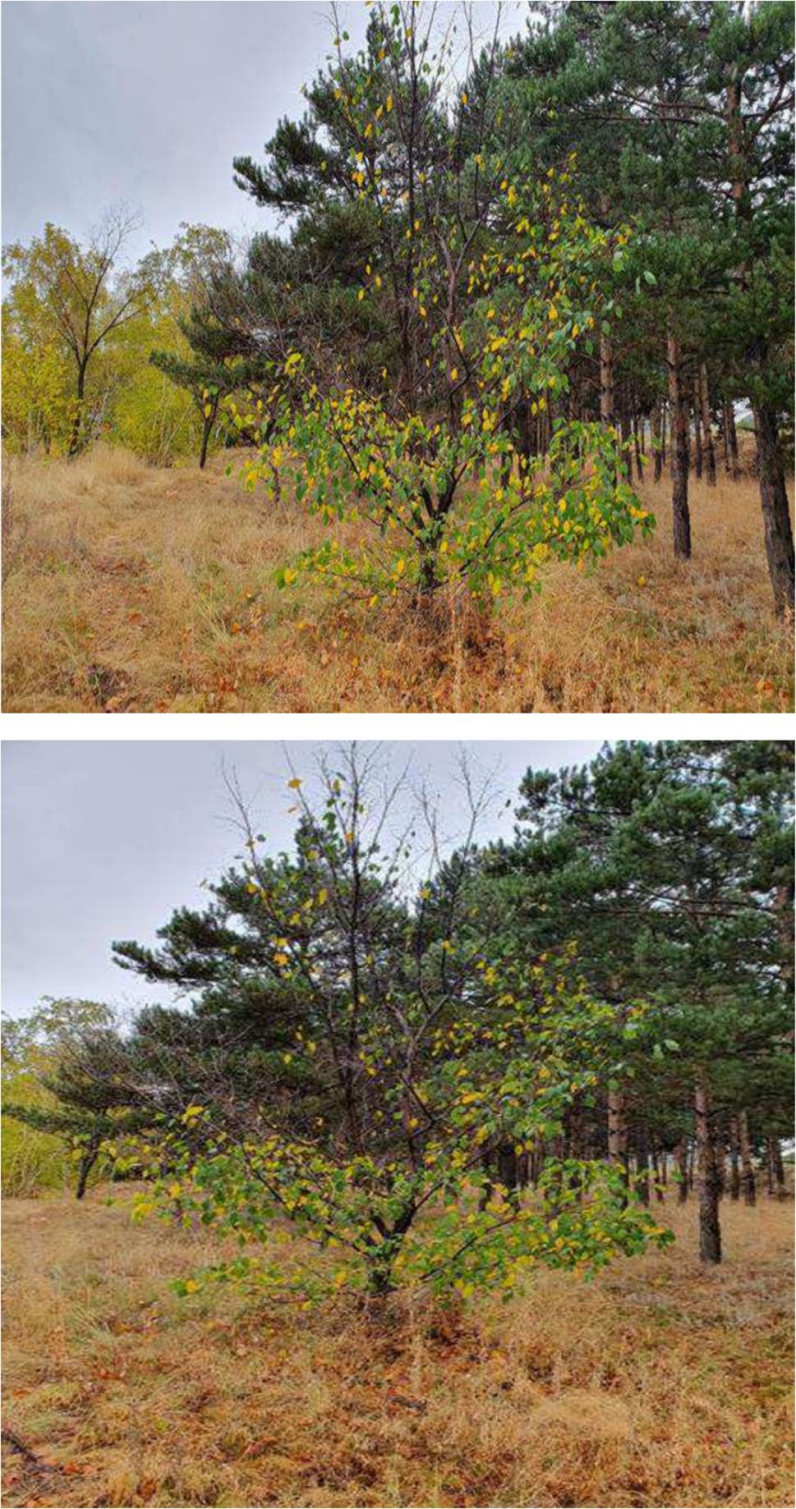
To regreen

In Fig. 8, the patient symbolically represented their body image through an illustration featuring a tree situated among other green trees, some of which displayed yellowed or fallen leaves while remaining upright. The patient elucidated that following mastectomy, alongside the removal of the breast, the loss of hair and eyebrows, and the physical weakness and fatigue experienced, they perceived themselves akin to the depicted tree—some leaves fallen, some turned yellow, yet endeavoring to remain standing. Notably, the patient’s emphasis on the verdant trees surrounding the depicted one underscored a comparison with its immediate environment, signaling an introspective reflection on their resilience.

Patient 6: From the outside, there is still me trying to stand, but I have changed a lot after this illness. I am like a tree trying to stand with these yellowing and falling leaves. After my breast was removed and treatment started, I felt like I was fading, just like the leaves of this tree. First, my breast was taken, then my hair and eyebrows fell, and the shape of my face changed. Of course, due to the side effects of the medication, the weakness, fatigue, and overall debilitation caused the leaves of this tree to yellow and fall even more.

**Fig. 9:**
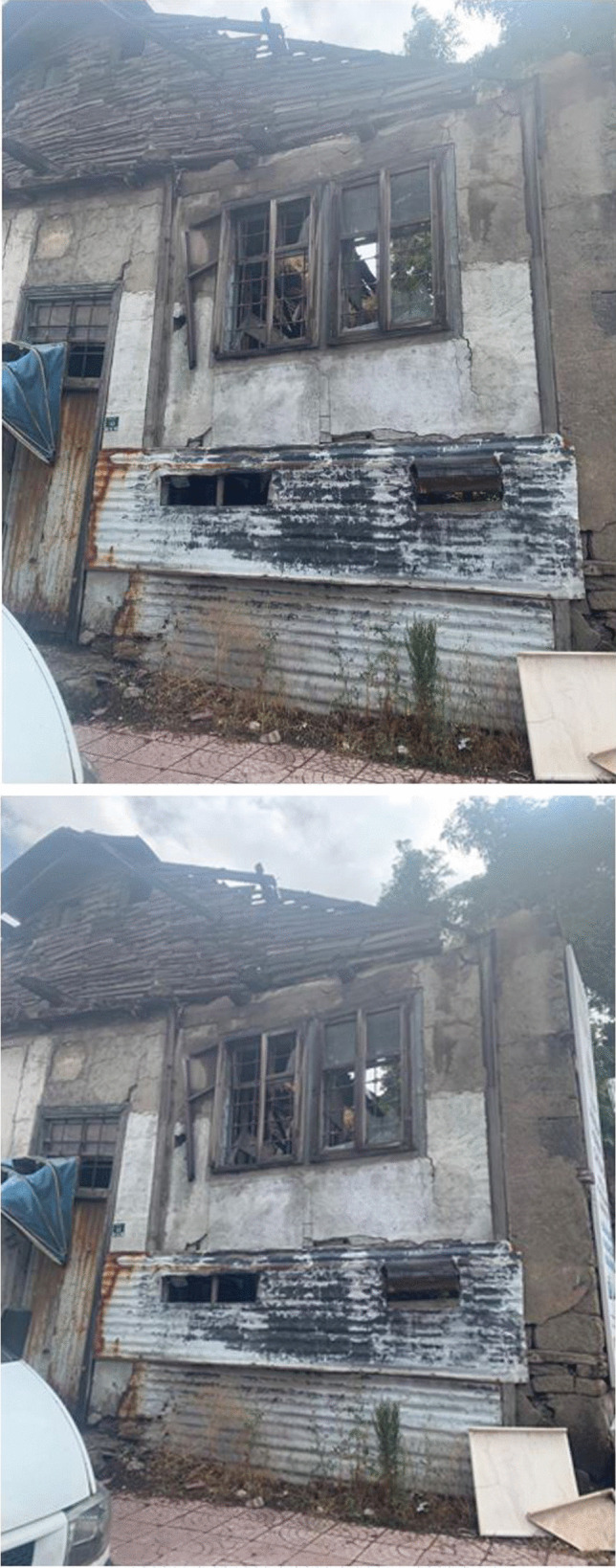
The destruction within me

In Fig. 9, the patient metaphorically represented their corporeal perception, likening it to a dilapidated domicile. Following a mastectomy, the patient articulated a sense of physical and psychological deterioration akin to the depicted dwelling’s state. They elucidated upon the manifold alterations in their physical form, attributing the disintegration to the removal of their breast, alopecia, and pervasive exhaustion. Moreover, the patient delineated the absence of familial support and the recurrent domestic discord, elucidating their deleterious influence on their emotional well-being. Emphasizing the pronounced impact of spousal and filial neglect, they underscored the exacerbation of their distress by this familial discord.

Patient 7: I feel like my body is collapsed, just like this house. But it is not just the illness that brought me down; not being able to get support from my family also shattered me. There is constant noise and arguments at home, and my children have distanced themselves from me even more. No one in the house comes to me and asks how I am. There is a woman who is both shattered by the disease and destroyed by not receiving social support, especially from her husband and children. Yes, the effects of the disease have affected me very badly. But the unrest at home affects me even more; I am collapsing day by day. No one comes to ask how I am at home; I can neither sleep properly nor eat well due to constant unrest and noise.

Patients who have undergone mastectomy shared their experiences through photovoice, revealing three main themes: isolation and coping challenges, the importance of psychosocial support and religious beliefs, and perceptions regarding external appearance, particularly feelings of loss and grief. Patients described their inclination to withdraw from crowded environments, preferring solitude, and encountering difficulties maintaining productivity and motivation. They expressed experiencing emotional distress, diminished hope, fatigue, and a tendency to avoid external stimuli, focusing inwardly on their struggles. Additionally, patients emphasized the crucial role of social support, family communication, and the significance of prayers and religious practices. Despite facing adversity, they held onto hope, focused on recovery, and displayed trust and acceptance in their journey. Finally, patients articulated feelings of loss, grief, an identity crisis, inadequacy, and reduced self-esteem. They struggled to reconcile their feminine identity due to changes in appearance, such as developing masculine traits post-mastectomy. The absence of breasts, along with hair loss during treatment, intensified their mourning and sorrow, leading to significant emotional distress. These insights shed light on the complex challenges faced by mastectomy survivors, highlighting the need for comprehensive support to address their physical, emotional, and psychological well-being.

## Discussion

Experiencing mastectomy, which involves the loss of a breast, can trigger a range of reactions in individuals, encompassing both favorable and unfavorable responses. Among the participants in this study, some embraced the alterations in their body shape following mastectomy with genuine acceptance and resignation, while others encountered challenges in accepting these changes. Numerous participants in the study indicated feeling tired, which led them to scale back their activities. They also noted experiencing hair and eyebrow loss, as well as physical discomfort like experiencing pain. These findings align with previous studies on the quality of life of Croatian women after mastectomy, which highlighted hair loss and fatigue as notable symptoms both 1 month and 1 year following the surgery [25]. Furthermore, following breast removal, some women expressed a desire to avoid social gatherings due to concerns about their appearance, and some women emphasized feelings of isolation as they perceived that those around them did not comprehend their circumstances. Similar studies conducted in different countries have also highlighted the widespread impact of mastectomy on women’s social lives [4, 26, 27, 28].

The formation of a positive body image in breast cancer patients is greatly impacted by encouragement from family members, especially children, spouses, and parents regarding their appearance. Despite undergoing mastectomies and losing one or both breasts, some participants did not feel ashamed of their current appearance. This lack of negative emotions was attributed to consistent positive support and acceptance from their families regarding their perceived imperfections. This observation is consistent with Doori et al. [29], who highlighted the connection between perceived social support and body image in women with breast cancer. When facing challenges due to changes in body shape, individuals often suffer emotional distress, potentially leading to anxiety, depression, and decreased self-esteem [27], Newlan and Greig [30]; [31]. Support from family and friends is vital in helping individuals feel valued and loved, facilitating their reintegration into society, and aiding in accepting their physical changes. The findings of this study suggest that body image encompasses individual beliefs, thoughts, feelings, and behaviors regarding one’s condition, a realistic understanding of one’s capabilities, and satisfaction with life and oneself.

In this study, individuals who had undergone mastectomy demonstrated their resilience by entrusting themselves to a higher power, accepting their circumstances, expressing gratitude, seeking healing, and experiencing relief once their ailment was addressed. These outcomes are consistent with the findings of [32], highlighting the significance of patience in navigating breast cancer. Patience offers advantages such as enhanced emotional regulation, a composed mindset, and subjective well-being. For mastectomy survivors, patience serves as a coping mechanism, enabling them to handle negative emotions and maintain a hopeful perspective, considering their life journey as part of a divine [33–36]. Maintaining optimism about recovery emerges as a crucial factor in the adaptation process for individuals confronting breast cancer post-mastectomy. Cultivating positive expectations regarding recovery is essential to prevent the exacerbation of their condition. This optimistic outlook plays a pivotal role in adapting to chronic illnesses like breast cancer. Participants in this study demonstrated optimism by engaging in prayer and worship alongside these efforts, seeking divine intervention for healing. Their expressions of hope centered on the aspiration to overcome breast cancer and resume their daily activities.

Interviews with most participants affirmed that the breast symbolized femininity, beauty, and motherhood. Previous studies have also echoed this sentiment, highlighting the breast as a vital aspect of womanhood [4]. Consequently, the loss of a breast due to mastectomy is often perceived as a significant blow to a woman’s identity and self-concept. Many participants in our study expressed a sense of loss and described feeling unwell or less feminine, even manly, after mastectomy. Existing literature underscores the female breast as a symbol of femininity and womanliness, with the loss of a breast viewed as a deprivation of womanhood or identity. Some participants mentioned struggling with household chores post-mastectomy, leading to feelings of emptiness and inadequacy. Similar studies have reported women experiencing difficulties in performing daily activities like shopping, cleaning, and hanging clothes post-mastectomy [37]. Inability to fulfill their previous roles in life post-mastectomy may trigger feelings of loss, affecting their sense of self and, consequently, their body image adversely.

Upon receiving their cancer diagnosis, participants expressed a spectrum of emotions, including shock, sadness, fear, and stress. The prospect of losing one or both breasts can also induce anxiety and depression aligning with Kubler-Ross’s theory of loss and grief, which delineates stages of denial, anger, and depression before acceptance. Participants vocalized their sadness regarding the diagnosis and exhibited nonverbal cues like tears and sorrowful facial expressions. Previous studies examining Kubler-Ross’s framework of psychological reactions in cancer patients have likewise delineated analogous stages of denial, encompassing emotions such as fear, surprise, a sense of normalcy, sadness, tears, resignation, and eventual readiness [38]. Following the recounting of their cancer diagnosis, some participants transitioned from tears to gradually adopting a composed demeanor as they discussed their current situation and their acceptance of both cancer and mastectomy. The majority of participants exhibited self-acceptance and a hopeful perspective, confident in their ability to recover. These results are consistent with earlier research on Kubler-Ross’s psychological responses in cancer patients, highlighting an initial stage of denial that progresses to acceptance, characterized by preparedness, optimism about treatment, and confidence in recovery [38].

## Limitations

Researchers have long acknowledged the adverse impact of mastectomy on a woman’s body image. However, our study’s findings, based on firsthand accounts from women themselves, underscore the trauma and profound sense of loss experienced by breast cancer patients post-mastectomy with the photovoice method. This study provides a more intimate and individualized understanding of body image, traditionally presented primarily through statistical analysis. It is hoped that this small exploratory study will contribute to enhancing supportive care concerning body image for women undergoing mastectomy.

With recent advancements in medical care and technology, an increasing number of women are surviving breast cancer. As a result, nurses must deepen their understanding of breast cancer treatments and their impact on women in their care. The findings of this study emphasize that experiences of body image after mastectomy are both unique to each individual and influenced by their circumstances, yet uniformly negative. Nurses and other healthcare professionals need to develop a more comprehensive understanding of how body image evolves among women following diagnosis and treatment. Instead of relying on broad assumptions based on factors like age or life stage, healthcare providers can benefit from each woman’s distinct and personal narrative regarding the significance of breast loss to her.

## Data Availability

No datasets were generated or analysed during the current study.
